# Linear combination test for gene set analysis of a continuous phenotype

**DOI:** 10.1186/1471-2105-14-212

**Published:** 2013-07-01

**Authors:** Irina Dinu, Xiaoming Wang, Linda E Kelemen, Shabnam Vatanpour, Saumyadipta Pyne

**Affiliations:** 1 School of Public Health, University of Alberta, Edmonton, Alberta T6G 1C9, Canada; 2 Department of Medicine, University of Alberta, Edmonton, Alberta T6G 2E1, Canada; 3 Department of Population Health Research, Alberta Health Services-Cancer Care, Calgary, Alberta, Canada; 4 Departments of Medical Genetics and Oncology, University of Calgary, Calgary, Alberta, Canada; 5 CR Rao Advanced Institute of Mathematics, Statistics and Computer Science, Hyderabad, India; 6 Public Health Foundation of India, Delhi, India

## Abstract

**Background:**

Gene set analysis (GSA) methods test the association of sets of genes with a phenotype in gene expression microarray studies. Many GSA methods have been proposed, especially methods for use with a binary phenotype. Equally, if not more importantly however, is the ability to test the enrichment of a gene signature or pathway against the continuous phenotypes which are routinely and commonly observed in, for example, clinicopathological measurements. It is not always easy or meaningful to dichotomize continuous phenotypes into two classes, and attempting to do this may lead to the inaccurate classification of samples, which would affect the downstream enrichment analysis. In the present study, we have build on recent efforts to incorporate correlation structure within gene sets and pathways into the GSA test statistic. To address the issue of continuous phenotypes directly without the need for artificial discrete classification and thus increase the power of the test while ensuring computational efficiency and rigor, new GSA methods that can incorporate a covariance matrix estimator for a continuous phenotype may present an effective approach.

**Results:**

We have designed a new method by extending the GSA approach called Linear Combination Test (LCT) from a binary to a continuous phenotype. Simulation studies and a real microarray dataset were used to compare the proposed LCT for a continuous phenotype, a modification of LCT (referred to as LCT_2_), and two publicly available GSA methods for continuous phenotypes.

**Conclusions:**

We found that the LCT methods performed better than the other two GSA methods; however, this finding should be understood in the context of our specific simulation studies and the real microarray dataset that were used to compare the methods. Free R-codes to perform LCT for binary and continuous phenotypes are available at http://www.ualberta.ca/~yyasui/homepage.html. The R-code to perform LCT for a continuous phenotype is available as Additional file 1.

## Background

Gene set enrichment analysis (GSEA) has greatly advanced high-throughput gene expression studies and a number of methods have been proposed to perform this type of analysis (see reviews by Goeman and Buhlmann [1] and Nam and Kim [2]). An important methodological challenge of GSA is the need to deal with large gene sets and small sample sizes. While most GSA methods employ a permutation-based approach to evaluate the significance gene sets, Kim and Volsky [3] gave a parametric view of the test statistic by assuming that the averages of fold changes across the gene-sets are distributed approximately normally. However, the majority of work in this field has focused on testing the enrichment of gene sets against binary, and sometimes categorical, phenotypes. Equally, if not more importantly, is the ability of the method to test the enrichment of a gene signature or a molecular pathway against a continuous phenotype. Such continuous variables are measured routinely and many important clinicopathological observations such as tumor size or the measurement of marker proteins are continuous. It may not always be technically easy or meaningful to categorize continuous phenotypes into two or more discrete classes. Indeed such artificial categorization may lead to inaccurate classification of the samples, which will eventually affect the downstream enrichment analysis.

We observed an important methodological distinction between the *competitive* and *self-contained* GSA approaches [1,4]. Competitive methods use gene permutation to test whether or not the association of the phenotype with a gene set is similar to its association with the other gene sets (the “Q1 hypothesis”), while self-contained methods employ sample permutation to test the equality of the two mean vectors of gene-set expressions which correspond to the two phenotype groups (the “Q2 hypothesis”). Here, we focused on the self-contained methods because, unlike the gene permutation approaches, sample permutation preserves correlations within gene sets; a property that we have used to design the proposed method for continuous phenotypes.

Correlations among gene expression measurements have long been observed, especially among measurements for functionally related gene set. Yet in the past, only the multivariate analysis of variance test for gene set analysis (MANOVA-GSA) for categorical phenotypes [5] and the Linear Combination Test (LCT) for binary phenotypes [6] have used a covariance matrix estimator of gene expressions to compute the enrichment test statistic. The main challenges in using these methods are the relatively small sample sizes and large gene sets; a situation which is not uncommon in GSA, especially in small microarray studies. To overcome these difficulties, shrinkage methods [7] have been used to estimate the gene expressions covariance matrix. However, GSA has rarely been used for continuous phenotypes, and currently no methods that incorporate a covariance matrix estimator are available. Previously, when we compared the performances of various self-contained GSA methods for binary phenotypes, we found that LCT was more computationally efficient than MANOVA-GSA and approximated its superior power very well. Here, we propose both an extension of LCT to continuous phenotype (hereafter referred to as LCT) and a modified version of LCT (hereafter referred to as LCT_2_). We compared the proposed methods with two existing GSA methods for continuous phenotype; namely, Significance Analysis of Microarrays for Gene Sets (SAM-GS) [8] and Global Test [9]. We used simulations to compare the performances of the GSA methods with small sample sizes and large gene sets.

In addition, we analyzed the performances of the GSA methods using real microarray gene expression data from prostate tumor samples of African-American prostate cancer patients [10]. Increased plasma or serum leptin levels have previously been found to be associated with development of prostate cancer [11-13]. We, therefore, used the C2 catalog, an extensive collection of metabolic and signaling pathways and gene sets, from the Molecular Signatures Database (MSigDB) of Gene Set Enrichment Analysis application of Broad Institute of MIT and Harvard. The catalog was screened for associations with human leptin gene (*LEP*) expression, a well-studied marker of adiposity, and various metabolic and inflammatory conditions, and we identified important molecular pathways that were associated with high expression of this marker in a prostate cancer cohort. In our comparative study, we focused on testing both the power and computational efficiency of the four GSA methods.

## Methods

### Linear combination test for a continuous phenotype

In this section we derive the LCT for a continuous phenotype. Our derivations follow the binary phenotype framework in that the correlation structure is accommodated in a similar way to the binary phenotype, and the shrinkage covariance matrix estimation is implemented to take care of the small sample size and large gene set problems.

Consider gene expression data consisting of a total of *n* subjects. The null hypothesis to be tested is, that the expression of a predefined gene set with *p* genes, {*X*_*1*_*, …, X*_*p*_} is not associated with the phenotype *Y*. One way of expressing this *multivariate* hypothesis *univariately* as a null hypothesis is *H*_0_; that is, no linear combination of *X*_*1*_*,…, X*_*p*_ is associated with the phenotype. Let *Z*(***β***) = *β*_*1*_*X*_1_ + *…* + *β*_*p*_*X*_*p*_ be a linear combination of *X*_*1*_*,…, X*_*p*_. Then, for a given vector ***β*** of combination coefficients, whether or not the combination *Z*(***β***) is associated with the phenotype can be tested in the univariate model as follows: *Y*_*i*_ = α_0_ + α_1_*Z*_*i*_*(****β****)* + *e*_*i*_, where *i* denotes subjects 1*, …, n* , *Y*_*i*_ denotes phenotype measurement of *i*^th^ subject, α_0_ and α_1_ are the intercept and slope respectively, and, *e*_*i*_ is *~ N(0,σ*^*2*^*)*. This expression describes a classical simple linear regression problem. To test *H*_0_, we can consider the most-significant linear combination of {*X*_*1*_*,…, X*_*p*_}; namely, the linear combination with the maximum sample correlation with the phenotype, among all possible linear combinations. We have

$$\beta *=\arg\;\max_{\beta }\;\rho_{Y,Z}^{2}{}_{\left(\beta \right)}$$

as the coefficients of the most-significant linear combination. As the square of the sample correlation, ignoring $\sigma_{Y}^{-2}$, we have:

$$\rho_{Y,Z}^{2}{}_{\left(\beta \right)}=\frac{\mathrm{Cov}{\left(Y,Z\left(\beta \right)\right)}^{2}}{\beta^{T}\hat{\Omega }\beta }.$$

Where $\hat{\Omega }\;$ is the gene expressions covariance matrix with the *hh*'– Th entry being

ωhh'=1n−1∑l=1,nxhl−x¯hxh'l−x¯h'

where *x*_*hl*_ is the gene expression corresponding to gene *h*, and subject *l*. Therefore,

$$\rho_{Y,Z}^{2}{}_{\left(\beta \right)}=\frac{\beta^{T}\mathrm{Co}v_{Y,X}\mathrm{Co}v_{Y,X}{}^{T}\beta }{\beta^{T}\hat{\Omega }\beta }.$$

Where Cov_*Y.X*_=*(Y,X*_*1*_*),…,Cov(Y,X*_*p*_*))*^*T*^ The above optimization problem can be written as

$$\beta *=\arg\;\max_{\beta }\frac{\beta^{T}\mathrm{A\beta}}{\beta^{T}\mathrm{B\beta}}.$$

Where ***A***=Cov_*Y,X*_Cov_*Y,X*_^*T*^ and $Β=\hat{\Omega }$. Thus, the solution to this optimization problem is the maximal eigenvector of *AB*^-1^ and $\rho_{Y,Z}^{2}{}_{\left(\beta *\right)}$ is the corresponding eigenvalue [14].

When the size of the gene set is larger than the sample size (i.e., *p > n*) the matrix ***B*** is singular. Similar to the adjustment implemented in MANOVA-GSA [5], a possible remedy for the singularity is to employ a shrinkage covariance matrix as proposed previously by Schafer and Strimmer [7]. Thus, the singular covariance matrix $\hat{\Omega }\;$ can be replaced with a shrinkage covariance matrix $\hat{\Omega }*$ given by ωhh'*=ρhh'*ωhhωhh' with shrinkage coefficients ρhh'*=1 if *h=h'*and ρhh'*=ρhh'min1max0,1−λ^', if *h≠h'* where *ρ*_*hh* '_ is the sample correlation between the *h–* th and *h'–* th genes, and the optimal shrinkage intensity ${\hat{\lambda }}^{*}$ can be estimated by λ^*=∑h≠h'Var(ρhh')/∑h≠h'ρhh'2.

The computational cost of incorporating the covariance matrix estimator into the test statistic in this way is very high. To address the computational efficiency problem, we use a group of normalized orthogonal bases, instead of the original observation vectors. First, we perform an eigenvalue decomposition of the shrinkage covariance matrix ${\hat{\Omega }}^{*}=\mathrm{UD}U^{T}$ (***V***_*1*_,..., ***V***_*p*_) = (***X***_*1*_,..., ***X***_*p*_)***UD***^− 12^ The square of the sample correlation can be rewritten as

$$\rho^{2}\left(\gamma \right)=\frac{\gamma^{T}\mathrm{Co}v_{Y,V}\mathrm{Co}v_{Y,V}{}^{T}\gamma }{\gamma^{T}\gamma }$$

where ***γ****=****D***^*1/2*^***U***^*T*^*β,* Cov_*Y.V*_=*(Y,V*_*1*_*),…,Cov(Y,V*_*p*_*))*^*T*^ According to a matrix algebra calculation [14], the coefficients of the most-significant combination are given by ***γ*** ∗ ∝ Cov_*Y*,***V***._ This LCT statistic is, therefore, proportional to the sum over the gene set of the square covariance between the phenotype and gene expression measurements, after the orthogonal transformation

$$\rho^{2}\left(\gamma *\right)=c\sum_{j=1,p}\mathrm{Cov}{\left(Y,V_{j}\right)}^{2}$$

where *c* is a constant. The statistical significance can be evaluated against the null hypothesis with a permutation test (permuting phenotype labels) using this test statistic. The constant *c* can be ignored in the permutation test. This approach is advantageous computationally because ${\hat{\Omega }}^{*}=\mathrm{UD}U^{T}$ is evaluated only once for the original data, and then there is no need to evaluate it for each permuted version of the data.

### A modification of the linear combination test for a continuous phenotype

We also considered an alternative form of LCT (LCT_2_) which we derived in the linear regression context. A least squares estimate of the regression function is given by $\hat{f}=X{\left(X^{T}X\right)}^{-1}X^{T}Y$ where ***X*** represents the gene expression matrix and *Y* represents the vector of phenotype values. In case of singularities, a shrinkage version of the regression function estimate analogous to LCT can be obtained. An alternative version of LCT, can be derived as the square of the *L*_*2*_ norm of the shrinkage regression function $\mathrm{LC}T_{2}=||\hat{f}|{|}_{2}^{2}$.

### Simulation study design

We carried out a number of simulation studies to compare the performances of the proposed LTC methods with two published self-contained GSA methods for continuous phenotype; namely, an extension of SAM-GS to continuous phenotype via regression analysis [8], and Global Test [9] which uses a random effects model to test the association between gene expression and phenotype. For each gene set of size *p*, we generated a gene expression matrix *X*_*nxp*_ We changed the number of observations *n* from 10 to 20, 50 and 100, and the gene set size *p* from 20 to 100, 200 and 400. We focused on scenarios where the gene set size is larger than the sample size, i.e. *p > n*, because these scenarios are more predominant and are challenging for GSA. We adopted a mixed correlation structure between genes in each set as follows: among the first *p*_*1*_ genes, the correlations are constant (ρ_ij_ = ρ); among the next *p*_*2*_ genes, the correlation between the i-th and j-th genes is ρ_ij_ = ρ^|i-j|^ with ρ = 0.0, 0.3, 0.6 and 0.9; and the remaining genes are not correlated. The various simulation scenarios are summarized in Table 1. For each gene set, a continuous phenotype was generated from a normal distribution N(**Xμ,*I****)*.where **μ** is a vector of length *p*. In the null model that we used to compare the size of the tests, we set **μ** to 0. In the alternative model that we used to check the power of the tests, first, we generated randomly five of the first 20 components of **μ** from *N (ν,|ν|)* with *ν* ranging from 0 to 2 in increments of 0.1, then, we generated randomly five of the next 20 components of **μ** from *N (−ν,|ν|)* with *ν* ranging from 0 to 2 in increments of 0.1; the rest remaining components were set at 0. The simulation data were replicated 1,000 times in each model. The p-values are based on 1,000 permutations.

**Table 1:** **Type I errors for four GSA methods: LCT, LCT**_**2**_**, SAM-GS and Global-Test**

**Type I**	**0.005**				**0.01**				**0.05**			
*n*	10				10				10			
*p*	20				20				20			
*p1 = p2*	5				5				5			
Method	ρ = 0.0	ρ = 0.3	ρ = 0.6	ρ = 0.9	ρ = 0.0	ρ = 0.3	ρ = 0.6	ρ = 0.9	ρ = 0.0	ρ = 0.3	ρ = 0.6	ρ = 0.9
LCT	.005	.003	.003	.002	.012	.012	.008	.009	.045	.049	.044	.052
LCT_2_	.006	.007	.004	.003	.013	.013	.009	.009	.048	.057	.048	.046
SAMGS	.004	.005	.006	.004	.014	.012	.007	.008	.044	.051	.048	.050
Global	.007	.008	.011	.011	.013	.019	.0117	.015	.053	.052	.054	.053
*n*	20				20				20			
*p*	100				100				100			
*p1 = p2*	20				20				20			
Method	ρ = 0.0	ρ = 0.3	ρ = 0.6	ρ = 0.9	ρ = 0.0	ρ = 0.3	ρ = 0.6	ρ = 0.9	ρ = 0.0	ρ = 0.3	ρ = 0.6	ρ = 0.9
LCT	.010	.009	.010	.010	.012	.014	.014	.016	.056	.053	.051	.058
LCT_2_	.011	.011	.009	.007	.014	.017	.017	.014	.056	.055	.055	.054
SAMGS	.010	.011	.010	.007	.012	.017	.018	.014	.056	.053	.052	.051
Global	.004	.004	.005	.006	.010	.005	.006	.012	.033	.041	.043	.052
*n*	50				50				50			
*p*	200				200				200			
*p1 = p2*	40				40				40			
Method	ρ = 0.0	ρ = 0.3	ρ = 0.6	ρ = 0.9	ρ = 0.0	ρ = 0.3	ρ = 0.6	ρ = 0.9	ρ = 0.0	ρ = 0.3	ρ = 0.6	ρ = 0.9
LCT	.010	.010	.010	.011	.016	.016	.015	.015	.057	.055	.051	.053
LCT_2_	.008	.010	.011	.008	.016	.016	.017	.013	.053	.058	.049	.059
SAMGS	.010	.008	.011	.009	.015	.015	.015	.019	.056	.057	.051	.050
Global	.004	.004	.004	.001	.009	.012	.012	.010	.040	.059	.052	.051
*n*	100				100				100			
*p*	400				400				400			
*p1 = p2*	60				60				60			
Method	ρ = 0.0	ρ = 0.3	ρ = 0.6	ρ = 0.9	ρ = 0.0	ρ = 0.3	ρ = 0.6	ρ = 0.9	ρ = 0.0	ρ = 0.3	ρ = 0.6	ρ = 0.9
LCT	.008	.006	.003	.005	.012	.010	.010	.007	.049	.046	.043	.034
LCT_2_	.009	.006	.004	.004	.012	.011	.011	.010	.055	.054	.042	.034
SAMGS	.006	.007	.005	.008	.012	.009	.010	.011	.047	.050	.047	.040
Global	.002	.003	.003	.002	.009	.005	.007	.006	.056	.036	.034	.034

The R-package to implement Global Test is available for download from http://www.bioconductor.org. The LCT tests and SAM-GS for continuous phenotypes were implemented by us using the R statistical software [15].

## Results

### Simulation study

**Figure 1:**
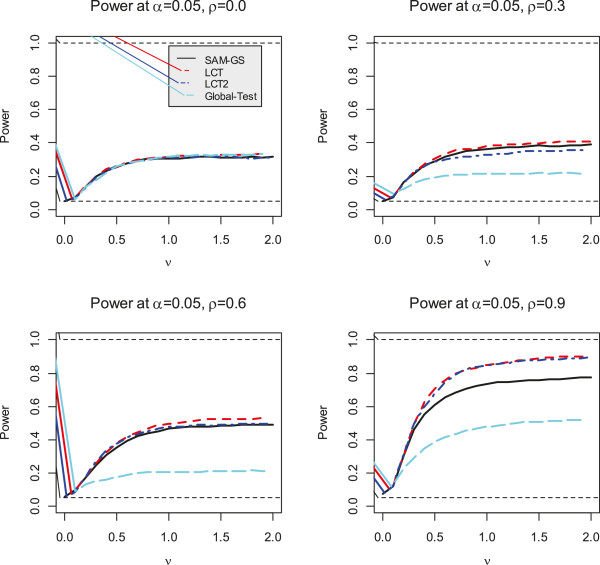
**Power comparison (*n*** **= 20 and *p*** **= 100) between four GSA approaches: LCT, LCT**_**2**_**, SAM-GS and Global Test.**

We found that the type I errors were similar across the four GSA methods (Table 1). As the sample size increased, the type I error moved closer to the nominal level, as is expected when permutation of phenotype labels is used. The empirical power (with *n* = 20 and *p* = 100) was calculated using a nominal level of 0.05 for values of the *ν* parameter ranging from 0 to 5 in increments of 0.25, and correlations between each pair of genes of ρ = 0.0, 0.3, 0.6 and 0.9 (Figure 1). When there was no correlation among genes (ρ = 0.0), the four GSA methods exhibited very similar testing powers. At low correlation values, the LCT_2_ method appeared to be conservative and less powerful; perhaps, because LCT_2_ is based on shrinkage of the regression function, similar to the ridge regression method [16]. However, with increasing correlations among genes (ρ = 0.3, 0.6, 0.9), the differences in power values between the LCT and Global Test methods became increasingly larger. Compared with either the SAM-GS or Global-Test methods, LCT and LCT_2_ both exhibited much better ability to deal with the given correlations among genes.

### Identifying gene sets associated with human leptin gene expression measurements

Leptin is a well-known marker protein for human adiposity and the circulating levels of leptin in the blood are directly proportional to the total amount of body fat. Leptin is also associated with various metabolic and inflammatory conditions. We applied all four GSA methods to analyze a real Affymetrix microarray dataset consisting of genome-wide transcriptomic measurements of prostate tumor samples from African-American prostate cancer patients [10] against the continuous phenotype of the human leptin gene (*LEP*) expression values. The publicly available data were downloaded from Gene Expression Omnibus [17] [GEO:GSE6956]. The data that we used in the present study are part of a larger microarray study into immunobiological differences in prostate cancer tumors between African-American and European-American men. Because *LEP* expression levels may be different between the two groups, we used only the data from the African-American group to test the LCT methods. For our analysis, we used the expression values of 13,233 genes measured in tumor samples from 33 patients. The tumor samples were resected adenocarcinomas from patients who had not received any therapy before prostatectomy and were obtained from the National Cancer Institute Cooperative Prostate Cancer Tissue Resource (CPCTR) and the Department of Pathology at the University of Maryland. According to Wallace *et al*. [10], the macro dissected CPCTR tumor specimens were reviewed by a CPCTR-associated pathologist who confirmed the presence of tumors in the specimens. The tissues were collected between 2002 and 2004 at four different sites. The median age of prostatectomy was 61 and the median prostate-specific antigen (PSA) at diagnosis was 6.1 ng/mL. Fifty-five percent of the tumors were stage pT2, and 45% were stage pT3 or more. Detailed RNA extraction, labeling and hybridization protocols were as described previously [10]. The gene expression values were centered and scaled across the samples before the four GSA methods were applied. The need for such standardization was pointed out in an earlier comparative study of GSA methods for a binary phenotype [18].

For comprehensive analysis, we used the C2 catalog from MsigDB [19] consisting of 1,892 gene sets, including metabolic and signaling pathways from major pathway databases, gene signatures from biomedical literature including 340 PubMed articles, as well as other gene sets compiled from published mammalian microarray studies. Following Subramanian *et. al*. 2005 [19], we restricted the size of gene sets to between 15 and 500 which gave us 1,403 gene sets for use in our analysis. Each gene set was tested for its association with the *LEP* expression measurements. A limitation of our study is that the findings come from a relatively small observational study and therefore cannot be generalized to other populations.

In terms of computational efficiency, we noted that LCT incorporated the covariance matrix into the estimations for only a small cost (CPU time of 413 seconds) compared with the cost using SAM-GS (CPU time of 397 seconds). In contrast, Global Test was very computationally attractive (CPU time of 92 seconds). The CPU times were recorded on our PC (Processor: x86 Family 6 Model 23 Stepping 10 Genuine Intel 3Ghz; 4GB RAM).

We compared the p-values for the gene sets obtained by the four methods; in particular, the lower p-values, which we assumed indicted the most interesting gene sets. Table 2 shows the percentages of the gene sets for which the p-values were less than 0.005, 0.01, 0.05, and 0.10 from the four GSA methods. We found that the performance of LCT and LCT_2_ was similar. The performance of SAM-GS and Global Test was also similar but different from the performance of LCT and LCT_2_, which is consistent with the results of the simulation study. To adjust for multiple comparisons when multiple gene sets are tested, false discovery rate (FDR) could be used instead of Type I error probability; however, the use of adjustment methods would not affect the conclusions of our comparative evaluation study. The FDR values were computed as described by Storey [20].

**Table 2:** Percentages of gene sets with p-values less than 0.005, 0.01, 0.05 and 0.10

**Method**	**P-value**			
	≤.005	≤.01	≤.05	≤.10
LCT	3	4	27	63
LCT_2_	3	4	20	46
SAM-GS	1	2	14	27
Global Test	0	2	15	34

Gene sets and pathways that were identified, by at least one of the four GSA methods, to be associated with the *LEP* gene expression measurements (p-value ≤ 0.05) are listed in Table 3 in ascending order of the p-values obtained using LCT. The corresponding FDR values were 0.195 for LCT, 0.197 for LCT2, 0.135 for SAM-GS, and 0.936 for Global Test. The adipocytokine signaling pathway was predicted to be strongly associated with *LEP* expression by all four GSA methods. This result was expected, given that adipocytokines are a group of adipose tissue-derived hormones that includes leptin. In addition to being linked to obesity and diabetes, adipocytokines may be involved in the regulation of angiogenesis and tumor growth [21]. Regulation of autophagy was found to be associated with *LEP* expression consistent with previous findings that leptin played a role in the neuroendocrine control of autophagy [22]. Autophagy is a fundamental process in tumorigenesis and treatment response because it can act as a tumor-suppression mechanism, yet it can also enable tumor cell survival under conditions of metabolic stress, including nutrient deficiency [23]. Furthermore, *LEP* expression was strongly associated with both hypoxia-inducible factor-1 (HIF1) targets (LCT p-value = 0.006; LCT_2_, SAM-GS and Global Test p-value <0.03) and the hypoxia pathway (LCT p-value = 0.035). Leptin can be activated in response to hypoxia in breast cancer cells where the process is mediated through hypoxia-inducible factor-1 [24,25].

**Table 3:** **Gene sets and pathways associated with *LEP *gene expression measurements**

**Gene set**	**Size**	**LCT**	**LCT**_**2**_	**SAMGS**	**Global**
NADLER_OBESITY_UP	46	0	0.004	0.108	0.098
HSA04920_ADIPOCYTOKINE_SIGNALING_PATHWAY	68	0.003	0.003	0.042	0.032
HSA04140_REGULATION_OF_AUTOPHAGY	26	0.004	0.007	0.003	0.002
HIF1_TARGETS	32	0.006	0.025	0.027	0.03
DORSEY_DOXYCYCLINE_UP	29	0.011	0.063	0.174	0.174
SHIPP_DLBCL_CURED_UP	28	0.013	0.003	0.01	0.02
JNK_UP	24	0.015	0.026	0.083	0.074
PROSTAGLANDIN_SYNTHESIS_REGULATION	28	0.016	0.04	0.165	0.155
CARDIACEGFPATHWAY	16	0.019	0.018	0.02	0.01
CITED1_KO_HET_UP	23	0.022	0.023	0.036	0.031
XU_CBP_DN	32	0.022	0.027	0.06	0.064
CHREBPPATHWAY	16	0.027	0.029	0.029	0.023
OXSTRESS_BREASTCA_UP	24	0.027	0.046	0.044	0.047
AGUIRRE_PANCREAS_CHR17	61	0.029	0.034	0.082	0.06
ST_GAQ_PATHWAY	27	0.031	0.047	0.109	0.083
HSA04340_HEDGEHOG_SIGNALING_PATHWAY	46	0.032	0.038	0.036	0.036
NFATPATHWAY	47	0.034	0.041	0.065	0.053
HYPOXIA_REVIEW	75	0.035	0.055	0.098	0.095
HSA04614_RENIN_ANGIOTENSIN_SYSTEM	16	0.04	0.108	0.087	0.076
CPR_NULL_LIVER_DN	16	0.041	0.047	0.038	0.036
HSA00380_TRYPTOPHAN_METABOLISM	49	0.043	0.055	0.11	0.1
HSA04630_JAK_STAT_SIGNALING_PATHWAY	135	0.045	0.065	0.173	0.167
DIAB_NEPH_UP	58	0.046	0.051	0.196	0.194
TRYPTOPHAN_METABOLISM	57	0.049	0.064	0.089	0.09
INSULIN_SIGNALING	93	0.049	0.068	0.187	0.18
PASSERINI_GROWTH	32	0.049	0.12	0.31	0.319
TNFA_NFKB_DEP_UP	18	0.05	0.07	0.152	0.169
FRUCTOSE_AND_MANNOSE_METABOLISM	24	0.055	0.02	0.039	0.041
ANDROGEN_AND_ESTROGEN_METABOLISM	21	0.058	0.028	0.046	0.042
POMEROY_DESMOPLASIC_VS_CLASSIC_MD_DN	38	0.091	0.048	0.13	0.116
TCA	15	0.101	0.058	0.042	0.037
HSA03050_PROTEASOME	21	0.103	0.113	0.051	0.048
PROTEASOME_DEGRADATION	27	0.122	0.082	0.028	0.029

The p-values from the four GSA methods, LCT, LCT_2_, SAM-GS and Global Test, are shown.

Among the gene sets and pathways associated with *LEP* expression only by the LCT method, we highlight the insulin signaling candidate pathway (LCT p-value = 0.049). A positive association between serum insulin levels and *LEP* expression has been reported in obese humans [26]. Furthermore, the association of circulating insulin-like growth factors with increased risk of prostate cancer has been reported in a meta-analysis [27]. Interestingly, the proteasome degradation candidate pathway was found to be significant by both Global Test (p-value = 0.029) and SAM-GS (p-value = 0.028), but not by LCT (p-value = 0.12). A small microarray study (N = 10) found that the genes in the proteasome degradation pathway were differentially expressed 72 hours after polyethylene glycol-leptin injection [28]. Other gene sets and pathways found to be significantly associated with *LEP* expression but with less well elucidated roles are shown in Table 3 and may be worthy of future investigation.

## Discussion

Many self-contained GSA methods have been proposed. However, although many of these methods have the potential to be generalized to any design, they have only been illustrated for a binary or categorical outcome. Thorough extension of these methods to a continuous phenotype has rarely been reported, and studies into their implementation, simulation studies to check type I error and power, and their application to real datasets are lacking. Here, we describe the extension of a “self-contained” GSA method from a binary to a continuous phenotype. The new GSA tests, LCT and LCT_2_, address several important technical issues. First, they provide a rigorous and computationally efficient approach to extend the enrichment test of a given gene set against a continuous phenotype. This will be of great help in studying a variety of informative measurements that cannot always be easily or meaningfully reduced to binary or categorical phenotypes. Second, because a pathway often consists of genes that are together involved in a biological mechanism or disease, gene expression levels within a pathway are expected to be correlated. Yet most traditional GSA methods fail to accommodate this important characteristic feature of gene expression datasets. While permutation methods using a valid test statistic can result in appropriate Type I error, the incorporation of a covariance matrix estimator into the test statistic is highly desirable because it often results in better power. Furthermore, we noted that when the gene set to be tested is larger than the sample size, the covariance matrix is ill-conditioned. To address this problem, a shrinkage method for covariance matrix estimation can provide a useful solution; however, shrinkage methods are rarely used in GSA, in spite of their implementation as an R-package which is free for download [7]. The computational cost of including a shrinkage covariance matrix estimator, especially for permutation-based hypothesis testing, can be very high. Notably in our LCT algorithm, we overcame this difficulty by using an orthogonal transformation of the gene expression matrix. In the LCT algorithm, therefore, the eigenvalue decomposition of the shrinkage covariance matrix is performed only for the original data, and not for the permuted versions.

We focused here on self-contained approaches and because competitive and self-contained methods test different hypotheses, it is important for the user to make an informed choice based on the hypothesis of interest and their understanding of the limitations of the two approaches (see reviews by Nam and Kim [2] and Dinu *et. al.*[4]). An important limitation of the self-contained approaches is that only a few genes can drive the association between the gene set and the phenotype. In such cases, post-hoc analysis can be used to reduce the gene set to a core sub-set associated with the phenotype. Such an analysis has been reported in simulations and in a real example for a binary phenotype [4].

The improvements that we have incorporated into our new GSA tests have given these tests a variety of advantages over the existing methods. We hope that they will be used for the rigorous testing of associations between different molecular pathways and gene signatures. At least of the measured clinic-pathological phenotypes are continuous. They include tissue features such as tumor size, staining based readouts; cellular characteristics such as the amount of lymphocytic infiltration in a tumor environment; and subject-specific measurements such as diagnostic or prognostic marker protein or metabolite concentrations. The LCT algorithm can adjust for continuous or categorical covariates following a regression framework. The *LEP* levels in the prostate tumor example that we considered may also have been influenced by patient-specific covariates such as body mass index (BMI), age, and/or smoking status. Smoking status did not show a significant association with *LEP* expression levels (p-value = 0.36), and BMI and age data were not available for our analysis.

To check the linearity assumption, exploratory data analysis should be used prior to running a formal inference. However, we noted that the small sample sizes that are common in microarray studies, would limit a thorough check for non-linearities. We also noted that the LCT method could be extended to accommodate non-linearities; however, a larger sample size would be needed. The simulations and real microarray studies which we conducted indicated that the LCT and LCT_2_ methods both performed very well for small sample sizes. The question of how small is small is debatable and depends largely on the study design. In the case of a binary/categorical phenotype, at least five samples per group are desirable. In the case of a continuous phenotype, assessing significance based on less than 10 samples is dangerous; an alternative would be to rely upon representations that are more descriptive/exploratory in nature. While LCT tests only linear associations between sets of genes and a continuous phenotype, SAM-GS and Global Test have been extended in a generalized linear model (GLM) framework and can accommodate multi-class, continuous, count, rate, and censored survival phenotypes. SAM-GS uses the sum of squares of the Wald statistic for individual genes constituting the pathway as the test statistic. Wald statistics are calculated as the ratio between the regression coefficient for an individual gene and its corresponding standard error. Global Test reduces the GLM to a random effects model, assuming the regression coefficients corresponding to the genes constituting the set are sampled from a common distribution with mean zero and constant variance. A score test statistic is used to test the null hypothesis of no association between the set and the phenotype. The SAM-GS and Global Test algorithms can both adjust for covariates, an attractive feature when accounting for other known prognostic factors in the screening of associations between gene sets and a phenotype.

## Conclusions

Our proposed LCT method for gene set analysis efficiently incorporates the gene expression covariance matrix into the test statistic. This approach has resulted in a powerful and computationally attractive method for testing the association of a given gene set with a continuous phenotype. Additional file 1.

### Availability and requirements

Project name: Linear Combination Test for Gene-Set Analysis of a Continuous Phenotype

Project home page: http://www.ualberta.ca/~yyasui/homepage.html

Operating system: Microsoft Windows XP.

Programming language: R 2.10.1.

## Abbreviations

GSA: Gene set analysis; LCT: Linear combination test; SAM-GS: Significance analysis of microarray for gene sets.

## Competing interests

The authors declare that they have no competing interests.

## Authors’ contributions

ID and XW developed the LCT methodology and designed/conducted the methodological study. SP, LEK and SV provided biological interpretations of the results of the analysis of the real-world dataset. ID, XW and SP drafted the manuscript which was critically reviewed and revised by all authors. All authors read and approved the final manuscript.

## Supplementary Material

Additional file 1 R code for the linear combination test (LCT) method for gene set analysis of a continuous phenotype. Click here for file
