# Supplementary material for: Linear combination test for gene set analysis of a continuous phenotype
Source: BMC Bioinformatics. 2013 Jul 1;14:212. doi: 10.1186/1471-2105-14-212 (PMC3717275; doi:10.1186/1471-2105-14-212)
Supplement: Additional file 1 — R code for the linear combination test (LCT) method for gene set analysis of a continuous phenotype. [file 1471-2105-14-212-S1.docx]

#****************************************************************************

# Title: R code for linear combination test for gene-set analysis of a continuous phenotype

#****************************************************************************

rm(list = ls())

library(corpcor)

library(qvalue)

GS.format.dataframe.to.list <- function(GS){

if(is.data.frame(GS)){

genes <- rownames(GS)

L <- NULL

for(ags in names(GS)){

w <- which(GS[,ags]==1)

if(length(w)>0) {

L <- c(L,list(genes[w]))

names(L)[length(L)] <- ags

}

}

L

}else{

GS

}

}

T2.like.SAMGS <- function(DATA, cl){

# DATA : expression data

# -> dataframe with rows=genes,

# columns=samples,

# weight: weights to T2 statistics of genes.

# cl : response vector for the samples

# IN THE SAME ORDER AS IN DATA

cl<-as.matrix(c(cl))

DATA<-as.matrix(DATA)

cl.DATA<-DATA%*%cl

sum(cl.DATA^2)

}

LCT <- function(GS, DATA, cl, nbPermutations=1000, silent=FALSE){

# GS : gene sets

# -> a dataframe with rows=genes,

# columns= gene sets,

# GS[i,j]=1 if gene i in gene set j

# GS[i,j]=0 otherwise

# OR

# a list with each element corresponding to a gene set = a vector of

# strings (genes identifiers)

#

#

# DATA : expression data

# -> a dataframe with rows=genes,

# columns=samples

#

# cl : response vector for the samples IN THE SAME ORDER AS IN DATA

#

# (1) pre-treatment of the gene sets and response vector

genes <- rownames(DATA) # gene names of the microarray data

nb.Samples <- ncol(DATA) # nb of samples

nb.GeneSets <- dim(GS)[2] # nb of gene sets

GS <- GS.format.dataframe.to.list(GS);

# change format of GS from dataframe to

# list

GS <- lapply(GS,function(z) as.numeric(which(genes %in% z)));

GS.sizes <- sapply(GS,length) # size of each gene set

# numericalized index of each GS

GS.data <- lapply(GS, function(z) as.matrix(DATA[z, ],ncol=nb.Samples));

# creat data of each GS (rows=genes,

# columns=samples)

GS.data <- lapply(GS.data,function(z) scale(t(z)));

# standardized genes in each GS

# (columns=gene, rows=samples)

cl=scale(cl) #standardized response

# (2) Eigen-decompsition of shrinkage pooled covariance matrix for each GS

Cov.Pooled<-lapply(GS.data, function(z) cov.shrink(z,verbose=FALSE, lambda.var=0));

# pooled covariance of genes in each GS

for (i in 1:nb.GeneSets){

EIGEN.decom<-eigen(Cov.Pooled[[i]]);

# eigen decomposition of pooled covariance for each GS

D<-EIGEN.decom$values; # shrinkag by adding a possitive constant s0

U<-EIGEN.decom$vectors;

GS.data[[i]]<-t(GS.data[[i]]%*%U)/sqrt(D)

# adjust data of each GS (rows=genes, columns=samples)

}

# (3) T-like stats obtained on 'true' data

sam.sumsquareT.obs <- sapply(GS.data, function(z) T2.like.SAMGS(z,cl))

# the T-like statistics obtained on 'true' data

# (4) stats obtained on 'permuted' data

sam.sumsquareT.permut <- matrix(NA,nbPermutations,nb.GeneSets)

for(i in 1:nbPermutations) {

ind <- sample(nb.Samples)

sam.sumsquareT.permut[i,] <- sapply(GS.data, function(z) T2.like.SAMGS(z[,ind],cl))

# SAMGS statistic for each gene set - for current permutation

if(!silent & i%%50 == 0)print(paste(i," permutations done."))

}

# (5) p-value and q-value

GeneSets.pval <- apply(t(sam.sumsquareT.permut) >= sam.sumsquareT.obs,1,sum)/nbPermutations

if(nb.GeneSets>=2){

GeneSets.qval <-0; #qvalue(GeneSets.pval)$qvalues

res <- as.data.frame(cbind("GS size" = GS.sizes,

"GS p-value" = GeneSets.pval,

"GS q-value" = GeneSets.qval ))

res <- cbind(res,"GS name"= names(GS))[c(4,1:3)]

}

if(nb.GeneSets==1){

#if there is only one set, no need to calculate q-value.

res <- as.data.frame(cbind("GS size" = GS.sizes, ##GeneSets.sizes,

"GS p-value" = GeneSets.pval))

res <- cbind(res,"GS name"= names(GS))[c(3,1:2)]

}

rownames(res)<-NULL

list("GS stats"=res)

}
